# The Criminal Justice Administrative Records System: A next-generation research data platform

**DOI:** 10.1038/s41597-022-01620-y

**Published:** 2022-09-12

**Authors:** Keith Finlay, Michael Mueller-Smith, Jordan Papp

**Affiliations:** 1 U.S. Census Bureau, Washington, DC USA; 2 University of Michigan, Department of Economics, Ann Arbor, MI USA; 3 University of Michigan, Institute for Social Research, Ann Arbor, MI USA

**Keywords:** Interdisciplinary studies, Law, Government, Databases

## Abstract

The Criminal Justice Administrative Records System (CJARS), a joint project of the U.S. Census Bureau and the University of Michigan, is a nationally integrated data infrastructure project designed to transform research and policymaking on the United States criminal justice system. At the University of Michigan, CJARS collects longitudinal electronic records from criminal justice agencies and harmonizes these records to track a criminal episode across all stages of the system. At the U.S. Census Bureau, harmonized criminal justice records can be linked anonymously at the person-level with extensive social, demographic, and economic information from national survey and administrative records.

## Introduction

In the United States, the social cost of crime is immense. In addition to the substantial costs of crime to victims, involvement in the criminal justice system not only has significant impacts on people accused or convicted of criminal offenses, but also on their families and communities. Yet there is no unified data infrastructure for measuring the U.S. criminal justice system, evaluating its policies, or understanding the population that interacts with it. The lack of data infrastructure reflects the highly decentralized structure of the criminal justice system, as data are held across thousands of disparate jurisdictions. There are important national data programs that cover criminal offenses or justice processes, such as the National Incident-Based Reporting System (NIBRS) and National Corrections Reporting Program (NCRP), but these programs do not allow us to understand how justice system processes are connected.

Comparable measures of criminal justice system performance require the integration of data from across the country. To identify effective policy levers, we must be able to connect criminal justice processes from arrest to sanction. Criminal justice data must be linked with other socioeconomic data to conduct dynamic benefit-cost analyses of spillover effects of criminal justice involvement. All of these types of linkages are rare, and integration with non-criminal justice data, such as labor market outcomes, is especially rare^1^. To address these shortcomings, the Criminal Justice Administrative Records System (CJARS) has undertaken the task of creating an integrated criminal justice data infrastructure that can be linked at the individual level across domains of the justice system and to non-justice system outcomes. The ultimate goal of CJARS is nationwide coverage of the major types of events that occur in the justice system (i.e., arrests, criminal court case filings, and terms of probation, incarceration, and parole).

CJARS is a data collection effort and dissemination platform founded in 2016 that aims to modernize research and statistical reporting on the U.S. criminal justice system. The project is a partnership between the University of Michigan and the U.S. Census Bureau. CJARS collects, harmonizes, and integrates administrative data from the five primary domains of the U.S. justice system: arrest, adjudication, incarceration, probation, and parole. The CJARS relational database schema parallels that organizational structure. A relational database is a collection of tables with rows and columns, where tables can be linked together using key variables common across tables. In the case of CJARS, tables represent event data from specific criminal justice processes and keys identify either unique people or criminal justice events processed by different agencies. Currently, CJARS includes over 2 billion lines of raw data that identify 175 million criminal justice events involving 37 million individuals across 30 states. The depth of historical data coverage varies by jurisdiction, but many states include series that extend back over 4 decades.

The U.S. lacks uniform rules across state and local jurisdictions on the privacy afforded to justice-involved individuals and what criminal justice contact is deemed public information^2^. Likewise, there is substantial heterogeneity in the development of data access mechanisms for researchers across the country. Lacking authority to compel data provision, CJARS implements a multi-pronged strategy for data acquisition. The team prioritizes data acquisition through data-use agreements, but data are also collected using public records requests, web scraping, bulk data downloads, and data donations.

The CJARS team harmonizes disparate data sources into a standard national data schema to facilitate data integration across jurisdictions and domains of the system. Entries in each of the five CJARS procedural databases reflect events relevant to the corresponding stage of the justice system: the arrest database is measured at the arresting charge level; the adjudication database is measured at the charge level; the probation, incarceration, and parole databases are measured at the level of terms of probation, incarceration, and parole, respectively. More information about the data schema and variables available in each relational database are available in the CJARS data documentation (https://cjars.isr.umich.edu/data-documentation-download). The CJARS data documentation also provides more details about data coverage by time and geography. In addition, the CJARS team is working on publicly available data tools to further assist users in assessing whether CJARS has sufficient data coverage for specific research or statistical applications.

**Fig. 1:**
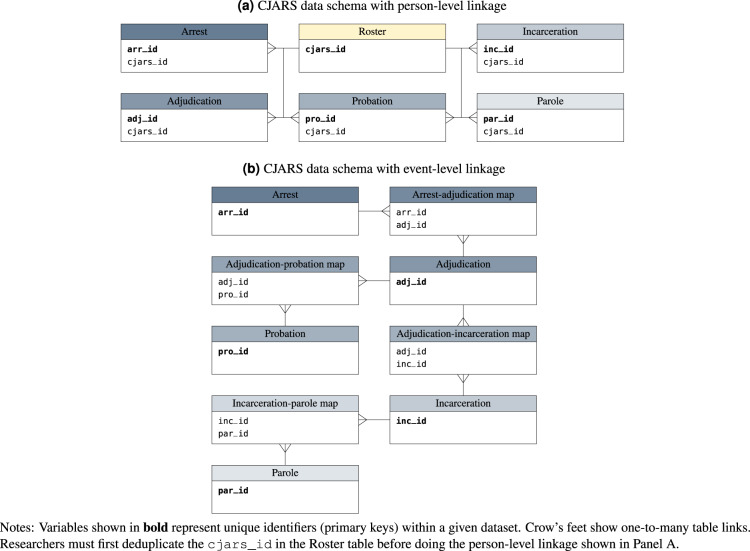
CJARS relational linkage structure of roster and procedural data files.

Figure 1 provides a visual representation of the organizational structure of the CJARS data infrastructure. Panel A of Fig. 1 shows the relational database structure, where there is one roster file and five procedural databases (one database for each major procedural domains of the justice system). The roster file contains a unique, anonymous identifier, the cjars_id, which identifies individuals within the data system. Each of the five procedural databases also contains the cjars_id to facilitate linkage across phases of the justice system, as well as an event identifier in each database (e.g., arr_id) that uniquely identifies events that occur within a given domain of the justice system. Panel B of Fig. 1 demonstrates how the procedural databases can be linked in conjunction with associative tables to reconstruct the sequence of events that are related to a single criminal episode. More information about the data schema and variables available in each relational database can be found in the CJARS data documentation.

CJARS data are available to qualified researchers on approved projects through the Federal Statistical Research Data Centers (FSRDCs), a network of 31 secure physical locations where CJARS can be linked to other anonymized survey and administrative data held in the Census Bureau’s Data Linkage Infrastructure. Researchers must apply and have projects approved before access, but the FSRDCs provide a proven, secure mode of data distribution that can safeguard the privacy and confidentiality of the extensive microdata contained in CJARS. It also provides the additional benefit of enabling individual level linkage with a range of other socioeconomic data held in the FSRDC network, including self-reported demographic characteristics, evolving family composition and place of residence, employment and earnings behavior, take-up of public benefit programs, and mortality. For example, CJARS can be linked at the individual level to responses from decennial census or American Community Survey (ACS) responses. The scope of the CJARS data holdings and other data available through the FSRDC network provide extensive opportunities for researchers to conduct novel research that previously was not possible without such a data infrastructure.

CJARS is continually expanding its geographic and procedural coverage through its data collection efforts. Continued data collection will provide broader coverage and thus improved capacity to support research. New vintages of the CJARS data infrastructure are made available in the FSRDC network on an approximately bi-annual basis.

## Results

Administrative data systems are not necessarily built for research or statistical purposes. During operational use, administrative records get updated, overwritten, or deleted. These operational changes may not be fully documented and so research or statistical users may have little guidance in how to process the data. Due to variation in data collection methods and the diverse set of solutions that the CJARS team uses to harmonize criminal justice records, there is a fundamental need to benchmark the data infrastructure against other available data series to both validate the strengths of CJARS and to highlight its potential weaknesses to interested researchers.

The degree of data integration available through the CJARS microdata is unprecedented, creating challenges for ideal technical validation testing. Our validation efforts focus mainly on reproducing available aggregate statistical series published by the Bureau of Justice Statistics (BJS), with the implicit assumption that success in matching aggregate information bolsters the validity of the underlying microdata contained in CJARS as well.

We evaluate CJARS against federal statistical series, such as the FBI’s Uniform Crime Reporting (UCR) program^3^ and several BJS programs: State Court Processing Statistics Series (SCPS)^4^, National Prisoners Statistics Program (NPS)^5^, National Corrections Reporting Program (NCRP)^6^, Annual Probation Survey^7^, and Annual Parole Survey^8^. These programs share with CJARS a common set of demographic and criminal justice measures in a common set of state and local jurisdictions with considerable historical data to assess data quality over time. Our focus is to benchmark CJARS data at the state-level, rather than to aggregate across all CJARS states whenever possible. This reflects the decentralization of the U.S. criminal justice system, and allows us to better assess our data collection and harmonization practices. Our evaluation of arrest data against the UCR can only be made at local and county levels, so we omit it here. Please see the CJARS benchmarking report (https://cjars.isr.umich.edu/benchmarking-report-download/) for these findings.

Our analyses focus on reproducing caseload count and flow estimates (e.g., yearly entries into prison as measured in the NPS), as well as caseload characteristics and outcomes (e.g., demographic characteristics of defendants in SCPS data) in CJARS-covered jurisdictions. CJARS-based estimates that closely corroborate existing federal estimates provide important evidence on the quality and accuracy of our nascent data infrastructure endeavor and the population-level, linkable microdata from which the CJARS-based estimates are constructed.

### Comparing CJARS adjudication to SCPS

The U.S. lacks a comprehensive statistical reporting program on the criminal court system. The closest option we have for validating CJARS adjudication records is the SCPS program, an occasionally produced statistical series that documents characteristics of felony defendants from large urban counties. A number of common caseload composition and case processing metrics can be calculated using both CJARS and SCPS, creating the opportunity to benchmark the CJARS adjudication data for the subset of records that overlaps with the definition of the scope of SCPS. This still provides useful information on gauging the quality of the algorithms applied to all of our data. Examples include average age of defendants, defendant gender and race/ethnicity, disposition type, time between disposition and sentencing, probation and incarceration sentence length, and offense type.

**Fig. 2:**
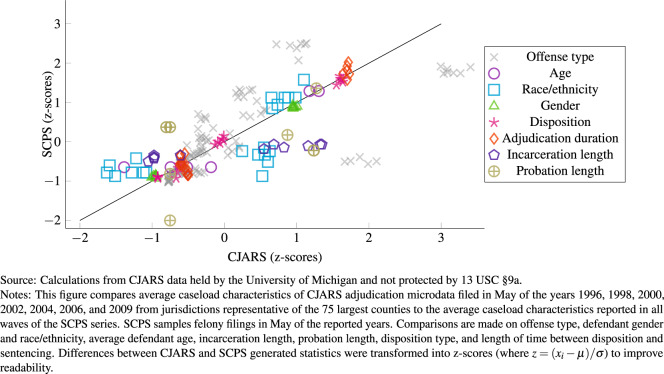
Standardized CJARS and SCPS-derived caseload statistics for felony defendants in large urban counties, by jurisdiction-year.

Figure 2 provides a scatter plot where comparable SCPS and CJARS statistics (e.g., average age of felony defendants) are plotted onto the y- and x-axes, respectively. Individual statistics are plotted for each of the 1996, 1998, 2000, 2002, 2004, 2006, and 2009 waves of SCPS with corresponding CJARS statistics built from the same corresponding time frames. The color/shape of a marker in the scatter plot represents a specific outcome and are repeated across the survey waves. The expectation is that the plotted points will cluster around the reference line, which has a slope equal to one. Clustering around the line indicates that the statistics generated using CJARS and SCPS are comparable.

Table 1 shows formal tests of the difference between means calculated using SCPS microdata and means calculated using CJARS case data. The means have been calculated over jurisdiction-year cells, which represents a total of 609 jurisdiction-year combinations covered by each wave of the SCPS data. In order to account for differential temporal and jurisdictional coverage, the tests first residualize for event-year by jurisdiction fixed effects, meaning the tests evaluate the statistical similarity of caseload means between SCPS and CJARS within overlapping jurisdiction-year cells. Since jurisdictions are not resampled within CJARS, we cluster our standard errors at the jurisdiction level to account for their repeated observation over time. When we evaluate the null hypothesis that SCPS and CJARS-based measures are equal in the weighted regressions, none of the empirical tests are statistically significant at the level of p < 0.05. For one metric (violent offenses), we do reject the null hypothesis at the level of p < 0.1 (p-value = 0.059).

**Table 1:** Testing differences of means of CJARS and SCPS-derived caseload statistics for felony defendants in large urban counties, by jurisdiction-year.

*Panel A: Defendant demographics*	Age	White	Black	Hispanic	Other race	Male	Female
Mean difference (SCPS-CJARS)	0.083	−0.081	−0.035	0.106	−0.0003	−0.018	0.018
	(0.263)	(0.098)	(0.044)	(0.095)	(0.003)	(0.017)	(0.017)
	[0.752]	[0.408]	[0.426]	[0.265]	[0.933]	[0.316]	[0.316]
	{0.677}	{0.468}	{0.553}	{0.256}	{0.729}	{0.215}	{0.215}
***Panel B: Offense type***	**Violent**	**Property**	**Drug**	**Public order**			
Mean difference (SCPS-CJARS)	0.096	−0.129	0.067	−0.034			
	(0.050)	(0.087)	(0.046)	(0.065)			
	[0.059]	[0.141]	[0.151]	[0.608]			
	{0.029}	{0.092}	{0.111}	{0.570}			
***Panel C: Disposition outcomes***	**Days between disposition and sentencing**	**Disposition:diversion**	**Disposition:dismissal**	**Disposition:conviction**	**Sentence:incarceration (months)**	**Sentence:probation: (months)**	
Mean difference (SCPS-CJARS)	−9.55	0.038	0.006	−0.026	−1.62	−4.40	
	(12.12)	(0.042)	(0.108)	(0.110)	(6.59)	(5.70)	
	[0.434]	[0.369]	[0.954]	[0.817]	[0.807]	[0.443]	
	{0.431}	{0.402}	{0.973}	{0.890}	{0.881}	{0.324}	

### Comparing CJARS incarceration to the NPS and NCRP

CJARS incarceration records can be compared to similar data from the NPS and the NCRP. All three data sources contain information that can be used to estimate annual prison entry counts, exit counts, and populations as well as incarceration rates. For succinctness, we present comparisons here only for annual entry counts.

**Fig. 3:**
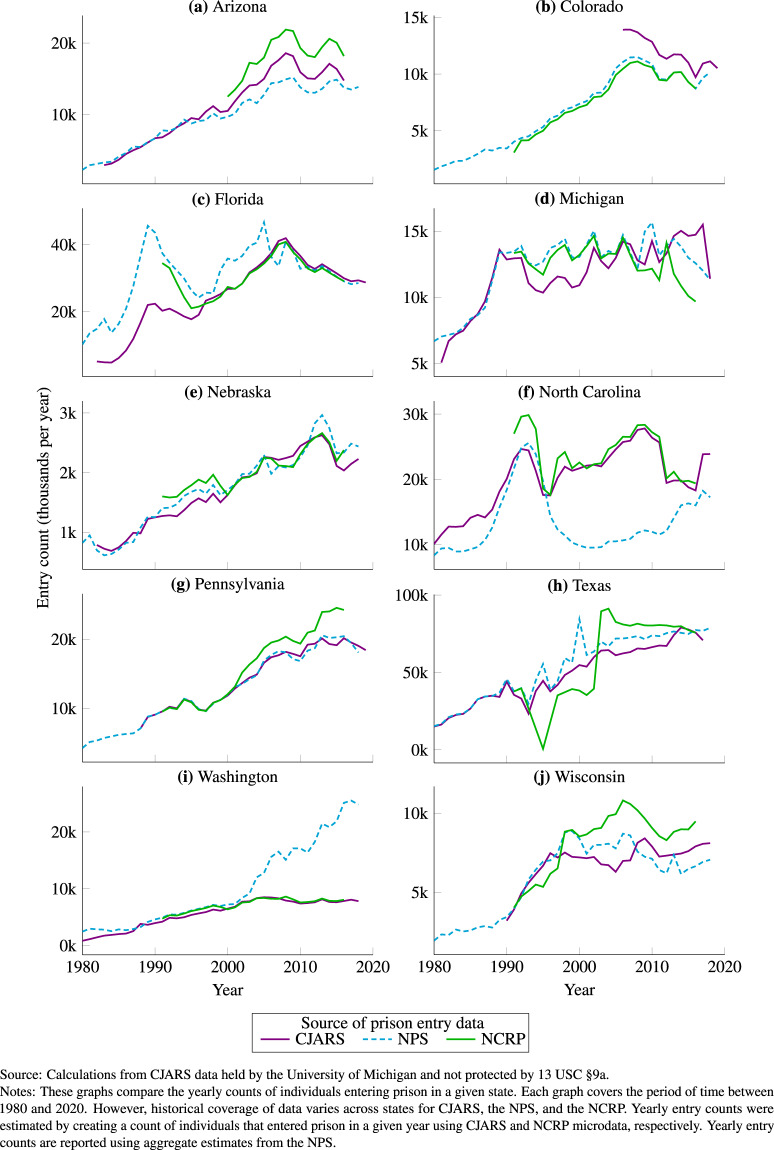
Comparison of CJARS, NPS, and NCRP-based estimates of annual incarceration entries, by state.

Figure 3 provides a comparison of annual entry counts as reported in the NPS and the NCRP, and from calculations using CJARS. A separate graph is given for each state for which CJARS has historical data holdings. In each graph, the purple line represents CJARS, blue the NPS, and green the NCRP. CJARS closely aligns with either the NPS, NCRP, or both in nearly every graph. For example, annual entry counts align well in Pennsylvania between all three data sources. Conversely, CJARS aligns better with either the NPS or NCRP but not both in, for example, Washington and North Carolina. A similar set of exercises that compare annual exit counts, year-end populations, and incarceration rates showing substantively similar findings can be found in the CJARS benchmarking report.

Additionally, we calculated the average absolute annual percent difference in the CJARS, NPS, and NCRP prison entry counts on a state-by-state basis. Across all years and states, CJARS entry counts differ from NPS and NCRP entry counts by an average absolute difference of 15.8% and 11.9%, respectively. In comparison, NPS and NCRP entry counts differ from each other by an average absolute difference of 16.1%–a larger discrepancy than CJARS has with either series.

### Comparing CJARS probation and parole to the annual probation and parole surveys

The probation information in CJARS provides information that can be compared to similar data from the Annual Probation Survey. Both CJARS and the Annual Probation Survey can be used to estimate yearly entry and exit counts, as well as yearly probationer populations and rates. Comparisons here focus on entry counts for succinctness.

**Fig. 4:**
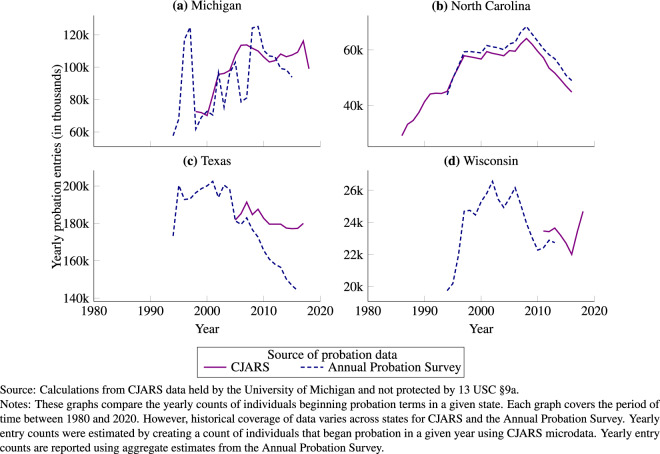
Comparison of CJARS and Annual Probation Survey-based estimates of annual probation entries, by state.

Figure 4 shows a comparison between probation entry counts observed in CJARS as compared to the Annual Probation Survey for each state where CJARS has historical data holdings. The graphs show substantial alignment in North Carolina. There also appears to be good alignment in Michigan, but there is considerable instability from year-to-year entry counts in the Annual Probation Survey, leading to large increases and decreases. In comparison, the CJARS data from Michigan provide much more stable counts from year-to-year. The graph for Texas in Fig. 4 shows similarities when coverage in the CJARS data begins (in the early 2000s). However, a gap forms over time in which more entries are observed in the CJARS data. A similar set of exercises that compare annual exit counts, year-end populations, and probationer rates showing substantively similar findings can be found in the CJARS benchmarking report.

Additionally, we calculated the average yearly percent difference on a state-by-state basis between CJARS and the Annual Probation Survey in terms of probation entry counts. Then, we calculated the average absolute yearly difference across all CJARS-covered states and years to quantify the average difference in entry counts between CJARS and the Annual Probation Survey. Comparing CJARS to the Annual Probation Survey shows an average absolute difference of 14.4%.

The parole information in CJARS provides information that can be compared against the same types of information gathered as part of the Annual Parole Survey. Both sources of parole data can be used to estimate yearly entry and exit counts, as well as yearly parolee populations and rates. Comparisons here focus on entry counts for succinctness.

**Fig. 5:**
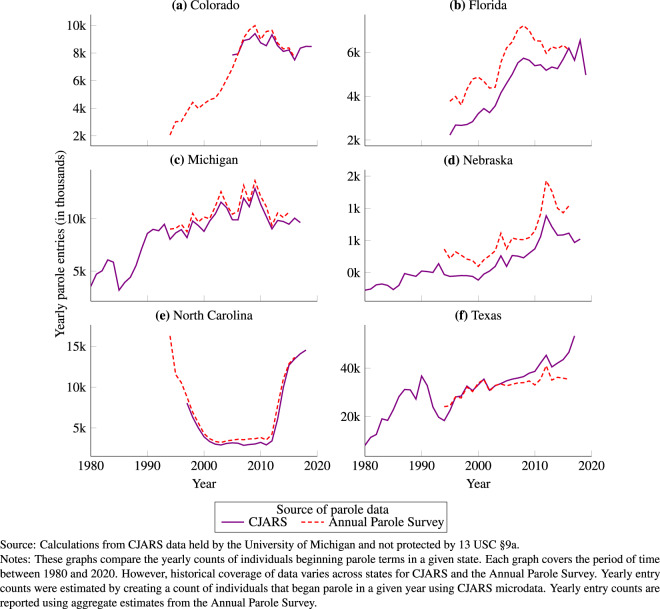
Comparison of CJARS and Annual Parole Survey-based estimates of annual parole entries, by state.

Figure 5 shows a comparison between parole entry counts observed in CJARS as compared to the Annual Parole Survey for each state where CJARS has historical data holdings. As can be seen in this figure, entry counts in CJARS and the Annual Parole Survey line up exceptionally well in almost all states. The one state where there is a slight difference is Nebraska, where the counts of events in CJARS are slightly lower than those reported in the Annual Parole Survey. However, the difference is consistent across years, and so the trends of changes in entry counts over time align between CJARS and the Annual Parole Survey. A similar set of exercises that compare annual exit counts, year-end populations, and parolee rates showing substantively similar findings can be found in the CJARS benchmarking report.

Finally, we calculated the average absolute yearly percent difference on a state-by-state basis between CJARS and the Annual Parole Survey in terms of parole entry counts. Then, we calculated the mean difference across all CJARS-covered states and years to quantify the average difference in entry counts between CJARS and the Annual Parole Survey. Comparing CJARS to the Annual Parole Survey shows an average absolute difference of 15.7%.

## Discussion

Given that CJARS data quality has been validated by closely replicating extant federal statistical series, the research data platform represents a transformative resource to advance knowledge on the determinants of criminal activity and the individual and community impacts of the U.S. criminal justice system. No existing repository available to researchers (1) is composed of records that cover criminal justice agencies of all types and from all geographies; (2) measures criminal justice events from arrest through sanction at the person level for the entire population; and (3) can be linked with extensive information about the socioeconomic characteristics and outcomes of justice-involved individuals.

**Fig. 6:**
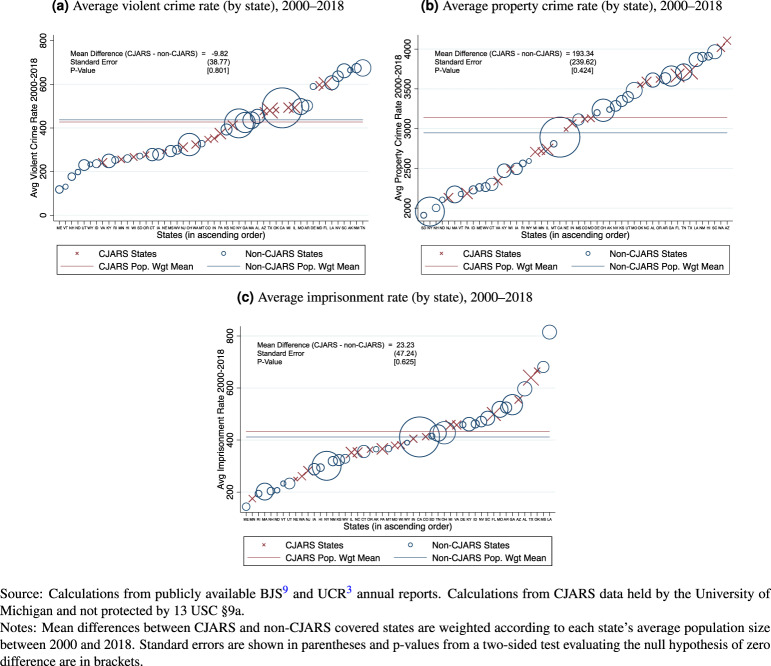
Representativeness of CJARS data coverage for UCR-measured crime rates and BJS-measured imprisonment rate.

CJARS data holdings continue to grow, but the data platform does not yet cover the entire country, raising questions about the appropriateness for population level statistics. Consequently, it is worth considering whether a CJARS dataset without complete national coverage is representative of the criminal justice system more broadly. In Fig. 6, we compare CJARS-covered states to non-CJARS covered states along three dimensions: average violent crime rates between 2000 and 2018^3^, average property crime rates between 2000 and 2018^3^, and average imprisonment rates between 2000 and 2018^9^.

From this exercise, we observe two key facts that bolster the case that CJARS can provide useful national estimates while continuing to grow toward national coverage. First, for each comparison series, the difference in the weighted means between CJARS and non-CJARS covered states is not statistically significantly different from zero. In fact, the differences in means are modest compared to weighted means observed in non-CJARS states: −2.2% for violent crime rates, 6.6% for property crime rates, and 5.6% for imprisonment rates. Second, we can also see in the figures that CJARS-covered states are represented throughout the distributions of each of the comparison measures, which suggests that CJARS data holdings can provide a representative perspective on a broad range of criminal justice processes.

## Methods

### Ethics

Justice-involved people are a vulnerable population, and a core principle of the CJARS project is that we acquire, store, and analyze criminal justice data securely and ethically so that the identities and characteristics of individuals in the CJARS data are kept confidential. The CJARS data collection and repository was reviewed and approved by the University of Michigan Health Sciences and Behavioral Sciences Institutional Review Board (approval number REP00000094); the review included additional oversight from a prisoner advocate per federal regulations. The project received a waiver for informed consent because it is built from existing electronic records maintained by government entities and involves no direct contact with or interventions applied to any human subjects. Any further research activity involving CJARS, including research conducted with anonymized CJARS data through the secure FSRDC network, requires separate Institutional Review Board approval from the researcher’s institution. The validation results described in this paper were separately approved as research activity (approval number HUM00208278).

### Data collection

The CJARS project collects data from police departments, sheriff offices, prosecutors, criminal courts, departments of corrections, and state criminal history repositories. The primary and preferred method of data collection is signed data-use agreements with agencies. These legal agreements delineate the responsibilities of the University of Michigan and the allowable uses of acquired data. Each of these agreements authorizes the University of Michigan to transfer data securely to the Census Bureau for linkage-based research and statistical work.

The CJARS team at the University of Michigan also collects data by submitting public records requests, sometimes referred to as Freedom of Information Act (FOIA) requests, in states where statutes require or allow agencies to make criminal history information available to the public. In a third approach, the University of Michigan harvests data that are publicly available online using web scrapers or bulk downloads. All web scraping is carried out under a set of ethical scraping policies to ensure that collection complies with agency website robots.txt and Terms of Use.

The goal of CJARS is to integrate data from federal, state, and local agencies. To maximize the growth of data coverage while using project resources efficiently, CJARS prioritizes acquisitions from agencies that manage statewide data systems, including departments of corrections, state court administrative offices, and state criminal history repositories. CJARS does acquire local agency data where the costs of doing so are low, such as where web scraping or public records requests are possible. As a benchmark, CJARS aims to spend no more than $0.01 per acquired row of data, which typically shifts our focus to larger jurisdictions where increasing returns to scale reduce per-observation acquisition costs.

### Data processing

**Fig. 7:**
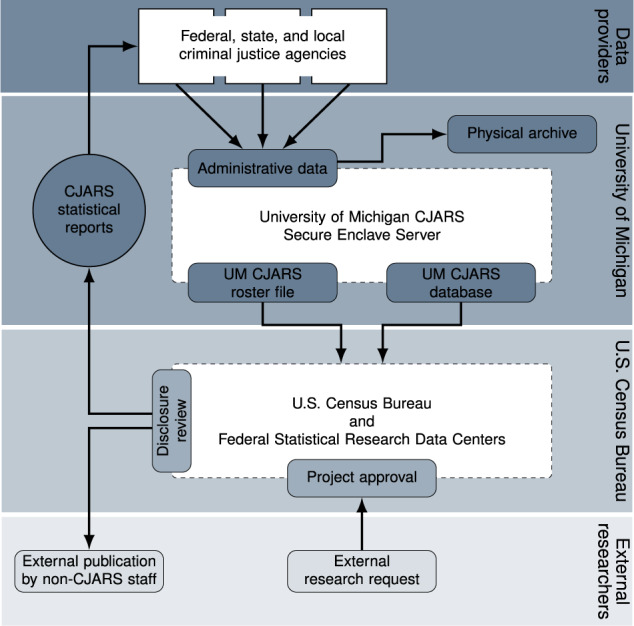
CJARS stakeholders, data exchange, record harmonization, and product development.

One of the major barriers to research on the criminal justice system is a lack of data integration across agencies. The CJARS team implements a systematic set of procedures to process and link the data it collects into a single, integrated data platform. Figure 7 provides a visual depiction of this process, which will be used to describe CJARS data processing in the following sections.

#### Data processing at the University of Michigan

Data collected from data providers are initially stored on secure data servers at the University of Michigan. Original data are cleaned and harmonized by the CJARS team at the University of Michigan. Cleaning and harmonization is an extensive process that involves transforming data received in its raw form from data providers into a format that fits the CJARS data schema. CJARS employs several strategies to conduct data processing and harmonization.

**Fig. 8:**
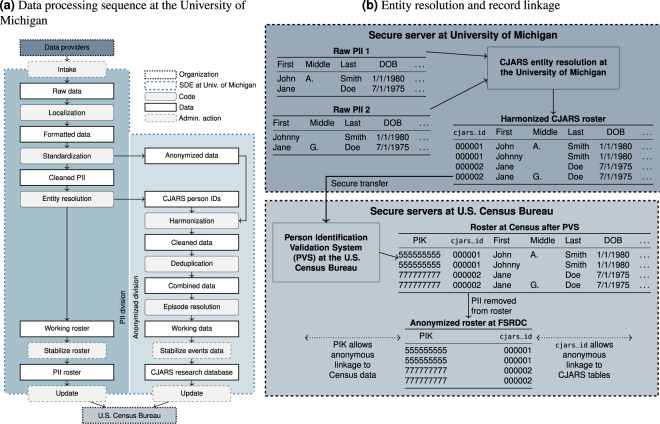
CJARS data processing steps.

CJARS data processing at the University of Michigan is broken out into a sequence of six steps (see Fig. 8). First, after data have been collected from a data provider, native data formats undergo *localization* to apply a common database format to each individual dataset. Second, the *standardization* stage extracts and harmonizes personally identifying information (PII), and imputes gender and race/ethnicity information where needed. PII variables are then used as inputs in *entity resolution* to generate a unique, person-level identifier that tracks involvement in the justice system across jurisdictions, over time, and through the various procedural domains of the justice system. Our approach to entity resolution leverages a biometrically trained probabilistic matching model (see https://cjars.isr.umich.edu/entity-resolution-download/).

The entity resolution process identifies and assigns each individual a unique cjars_id (see Fig. 8). These identifiers are added to the cleaned data that have been stripped of all PII variables and are transferred to the anonymized partition of the CJARS secure data servers, where the records undergo further processing. The separation between the PII and anonymized partitions aims to restrict access to PII variables to CJARS staff with an operational need, adding additional privacy and confidentiality protections within our organization.

With cjars_ids attached, the data next proceeds through *harmonization*, which brings each individual dataset into the common national schematic adopted in the CJARS data platform. The purpose of harmonization is to align disparate source files to reduce barriers for multi-jurisdictional research. This is accomplished through both populating a uniform set of variables from each source file and ensuring coded values follow a consistent standard. One example of the latter is offense classification, where we have to translate over 4 million unique text descriptions into a unified set of offense codes. This specific task is accomplished through a machine learning model that CJARS has developed in partnership with Measures for Justice, known as the Text-based Offense Classification (TOC) tool (see https://cjars.isr.umich.edu/offense-classification-download/).

The final two data processing steps at the University of Michigan involve *event deduplication* and *episode resolution*. Because we receive data from agencies with overlapping data coverage (e.g., statewide repositories and local criminal courts) as well as repeated extracts over time with evolving information on local caseloads, the deduplication stage is critical to ensure that we are not over-counting the number of distinct points of contact that individuals have with the justice system. Lastly, episode resolution generates crosswalks that connect the procedural stages of the justice system to each other using contextual information like event timing, offense types, and sentencing outcomes.

#### Data integration at the Census Bureau

After data processing at the University of Michigan is complete, the data are securely transferred to the U.S. Census Bureau for integration into the FSRDC system. This begins with the CJARS roster file being processed through the Person Identification Validation System (PVS)^10^, a probabilistic record linkage system that generates a crosswalk between the cjars_id and the Protected Identification Key (PIK). PIKs uniquely and anonymously identify individuals in the U.S. within the FSRDC system, and allow researchers to link de-identified CJARS and non-CJARS survey and administrative records at the individual level. Research occurs within a secure computing environment that is available at Census Bureau headquarters and in the FSRDCs across the U.S.

## Data Availability

CJARS data may be accessed through the FSRDC network, which is composed of 32 secure physical locations where qualified researchers on approved projects can link anonymized data from the Census Bureau’s Data Linkage Infrastructure. Many FSRDC projects are currently approved for virtual access. All five CJARS procedural databases and the cjars_id-to-PIK crosswalk file (also known as the anonymized roster file) are available to qualified researchers with approved Census Bureau projects in its FSRDC network. Data are only accessible through the FSRDC network to ensure the privacy and security of the sensitive records contained in CJARS. Anonymized data are stored in SAS format, although the FSRDC network has a wide range of statistical software available to support researchers working with their preferred tools. The CJARS team has produced extensive documentation and support tools to assist data users. The CJARS data documentation provides information on project scope, data collection methods, data coverage, data access and security, data processing and harmonization, linkage techniques, and data providers. The documentation explains the data schema and includes a variable codebook with variable-level descriptive statistics and data notes describing unique aspects of specific data acquisitions that data users should consider. Prospective researchers can apply to use CJARS through the FSRDC project application process, which begins by speaking with an FSRDC administrator. The CJARS team has produced a proposal guide that walks researchers through the process, identifies who can apply, describes the criteria used to approve projects, and documents data-use limitations (see https://cjars.isr.umich.edu/proposal-guide-download). The list of FSRDC locations can be found at https://www.census.gov/about/adrm/fsrdc/locations.html.

## Code availability

CJARS project code that does not contain sensitive information is made available at https://github.com/umcjars. This includes a template of our data production process, which can be found at https://github.com/umcjars/cjars_production_code_shell. In addition, code for the validation exercise presented in this article can be found at https://github.com/umcjars/cjars_bjs_validation. Code used for other research projects involving CJARS data conducted at the Census Bureau through the FSRDC system is available upon request via email at erd.cjars@census.gov. This code must be cleared first by the Census Bureau to avoid disclosure of any potentially sensitive information.
